# Urinary β2-microglobulin and disease activity in patients with tubulointerstitial nephritis and uveitis syndrome

**DOI:** 10.1186/s12348-018-0166-3

**Published:** 2018-12-29

**Authors:** Lorraine M. Provencher, Aaron M. Fairbanks, Michael D. Abramoff, Nasreen A. Syed

**Affiliations:** 1 Department of Ophthalmology and Visual Sciences, University of Iowa Hospitals and Clinics, 200 Hawkins Drive, Iowa City, IA USA; 2 The Stephen A. Wynn Institute for Vision Research, University of Iowa, 375 Newton Road, 4111 MERF, Iowa City, IA USA; 3 Iowa City Veterans Affairs Medical Center, 601 Hwy 6 West, Iowa City, IA USA; 4 Department of Pathology, University of Iowa Hospitals and Clinics, 200 Hawkins Drive, Iowa City, IA USA

**Keywords:** β2-microglobulin, Tubulointerstitial, Nephritis, Uveitis, TINU

## Abstract

**Background:**

Urinary β2-microglobulin (Uβ2M) is elevated in tubulointerstitial nephritis and uveitis (TINU) syndrome and has emerged as an important diagnostic tool. This study aims to determine whether Uβ2M correlates with uveitis activity in TINU.

**Methods:**

Retrospective observational case series of nine patients with TINU and ≥ 30 days follow-up. Presenting symptoms, visual acuity, uveitis characteristics, follow-up, Uβ2M, serum creatinine (SCr), urinalysis, and renal biopsy results were collected.

**Results:**

A correlation between Uβ2M and anterior chamber (AC) cell (*r* = 0.69, 95% CI 0.46–0.84), flare (*r* = 0.65, 95% CI 0.39–0.81), trended toward a stronger correlation than SCr and AC cell (*r* = 0.59, 95% CI 0.29–0.79), flare (*r* = 0.52, 95% CI 0.19–0.75). Uβ2M decreased over 1–2 years while SCr returned to normal within a few months.

**Conclusions:**

Uβ2M correlate with uveitis activity and trend down over the course of TINU. Uβ2M may serve as a useful tool in determining where patients are in their systemic disease course.

## Background

Tubulointerstitial nephritis and uveitis (TINU) syndrome is a rare, idiopathic inflammatory disorder characterized by acute tubulointerstitial nephritis (TIN) and uveitis without an otherwise known systemic cause [1, 2]. Typical ocular involvement is symptomatic, bilateral anterior uveitis [3], though cases of intermediate [4] or panuveitis with chorioretinal lesions [5] have been reported. Conversely, symptoms of TIN are non-specific (e.g., fever, malaise), often mild, and may precede (65%), follow (21%), or coincide (15%) with the onset of ocular symptoms [2]. TINU syndrome represents only 1–2% of patients at tertiary uveitis centers [6, 7], a likely underrepresentation given the difficulty in recognizing acute TIN without appropriate laboratory testing [8]. TIN in TINU syndrome tends to be self-limited with gradual normalization of renal function, i.e., serum creatinine (SCr). However, uveitis in TINU syndrome is often chronic and relapsing. Improved management of TINU syndrome may minimize sequelae of chronic uveitis and the discomfort associated with symptomatic recurrence of the uveitis.

Definitive diagnosis of TIN requires renal biopsy. However, this invasive procedure carries rare but significant risk of severe or life-threatening hemorrhage. Urinary β2-microglobulin (Uβ2M), which is elevated in 88–100% of reported cases of TINU syndrome, has emerged as a useful diagnostic tool in TINU syndrome [2, 8, 9]. The landmark Mandeville diagnostic criteria, detailed in a systematic review by Mandeville, Levinson, and Holland in 2001 [2], incorporated Uβ2M as part of evidence for interstitial nephritis. In healthy individuals, Uβ2M is a small protein excreted by the renal glomeruli into the ultrafiltrate, with 90% resorbed by the tubules. In acute TIN, reabsorption of Uβ2M is compromised and Uβ2M becomes elevated in the urine [10]. In TINU syndrome, Uβ2M is typically highly elevated and may remain elevated for months, long after laboratory tests of renal function [SCr or urinalysis (UA)] have normalized [2, 11].

TINU syndrome, with systemic manifestations and laboratory abnormalities, is an inflammatory condition where an indicator of overall disease activity would be valuable in assisting therapeutic decision-making. Though slitlamp findings are typically used to titrate therapy for anterior uveitis, it is difficult to know the activity level of the patient’s systemic inflammation and when and if the uveitis will recur after therapy is tapered or discontinued, especially if the eye is quiet on treatment. Traditional markers of systemic inflammation (i.e., sedimentation rate, c-reactive protein) are non-specific and often normal. This study aims to determine whether repeated laboratory analysis of Uβ2M levels, or other tests of renal function, correlates with the activity of uveitis over time in TINU syndrome.

## Methods

We conducted a retrospective chart review in the eye clinic at the University of Iowa Hospitals and Clinics from May 1, 2009, to February 15, 2017. This study was approved by the Institutional Review Board, adhered to the tenets of the Declaration of Helsinki, and was compliant with the Health Insurance Portability and Accountability Act.

We selected all patients from the electronic health record (EHR), Epic (Epic Inc., Madison, WI) during the study period with a diagnosis of “iridocyclitis” according to International Classification of Disease codes version 9 (ICD-9) and ICD-10 via an EHR search. ICD-9 codes (Jan. 1, 2009–Oct. 1, 2015) included acute and subacute iridocyclitis, unspecified (364.00), primary iridocyclitis (364.01), recurrent iridocyclitis (364.02), secondary iridocyclitis, noninfectious (364.04), chronic iridocyclitis, unspecified (364.10), unspecified iridocyclitis (364.3). ICD-10 codes (Oct. 1, 2015–Feb. 15, 2017) included primary iridocyclitis (H20.01), unspecified acute and subacute iridocyclitis (H20.00), unspecified iridocyclitis (H20.9), and chronic iridocyclitis (H20.12).

From the patients identified by the EHR search, an EPIC-based laboratory data search for all patients with an elevated Uβ2M (> 160 μg/L) was performed. From these patients, those with a clinical diagnosis of TINU syndrome made by a uveitis specialist were selected for study. Additional inclusion criteria were follow-up of at least 30 days.

### Clinical data

The following clinical data were collected: initial ocular symptoms, systemic symptoms associated with TINU syndrome, presenting visual acuity (in logarithm of the minimum angle of resolution [logMAR]), intraocular pressure (IOP), uveitis activity (SUN grading [12]), treatment, uveitis relapse or exacerbation, and days of follow-up. Any history of diabetes was noted. An exacerbation was defined as increased inflammation during steroid taper that required escalation of therapy. Relapse was defined as reappearance of anterior chamber inflammation after a minimum of 2 weeks following taper from any topical or systemic immunomodulatory medications. Remission was defined as inactive disease for at least 3 months after discontinuation of all treatment [12].

### Laboratory data

Antinuclear antibody (ANA), cytoplasmic and perinuclear anti-neutrophil cytoplasmic antibody (c- and p-ANCA), angiotensin-converting enzyme (ACE), rheumatoid factor (RF), syphilis serology, Lyme disease serology, HLA-B27, Uβ2M, SCr, urinalysis (UA), and any renal biopsy results were collected. Uβ2M levels greater than 160 μg/L were considered elevated. The initial Uβ2M and any subsequent Uβ2M measurements were collected. The Uβ2M elevation at each time point was calculated as a ratio of the Uβ2M level at that time point to the maximum Uβ2M recorded for that patient and expressed as a percentage. SCr was considered elevated if > 0.9 mg/dL in patients less than 16 years of age. For males and females older than 16, SCr was elevated if over 1.2 and 1.0 mg/dL, respectively. Patients were categorized based on Mandeville criteria [2].

### Data analysis

Pearson correlation was calculated between Uβ2M elevation (%) and uveitis activity (anterior chamber cell, flare, and anterior vitritis) and SCr and uveitis activity. Statistical analysis was by Prism software (version 7.0a, GraphPad, La Jolla, CA). A 95% confidence interval (95% CI) was calculated to accompany mean data.

## Results

The EHR diagnosis search resulted in 1283 patients. Of these patients, 30/1283 had an elevated Uβ2M. Two of 30 were excluded for error in diagnosis code (no chart documentation of uveitis), and 7/30 did not meet follow-up criteria. Of the remaining 21/30 patients, 12/21 were excluded for a probable alternative cause of their uveitis or elevated Uβ2M: herpetic iridocyclitis (3), +HLA-B27 (3), + RF (2), highly elevated ACE (1), history of chronic kidney disease (1), IgA nephropathy (1), and history of recurrent/recent kidney stones (1). The remaining 9/30 patients had a clinical diagnosis of TINU syndrome and met criteria for full analysis.

### Demographics and clinical characteristics at presentation

The mean age at diagnosis was 33.4 years (range 12–67, 95% CI 15.8–51.1) and 56% of patients were female. All patients had ocular symptoms (Table 1); 33% had systemic symptoms. Mean logMAR visual acuity was 0.12 (95% CI 0.04–0.21) and mean IOP was 14.6 mmHg. All patients ultimately had bilateral uveitis, and 67% had primarily anterior uveitis (Table 1).

**Table 1:** Uveitis symptoms, laterality, onset, and location at presentation

	No. of patients (%)
Symptoms at presentation	
Redness	8 (89%)
Photophobia	7 (78%)
Decreased vision	6 (67%)
Pain	6 (67%)
Tearing	5 (56%)
Floaters	3 (33%)
Laterality and onset	
Bilateral	9 (100%)
Sequential involvement	6 (67%)
Primary location of uveitis	
Anterior	6 (67%)
Intermediate	2 (22%)
Panuveitis	1 (11%)

### Laboratory trends and uveitis activity

**Fig. 1:**
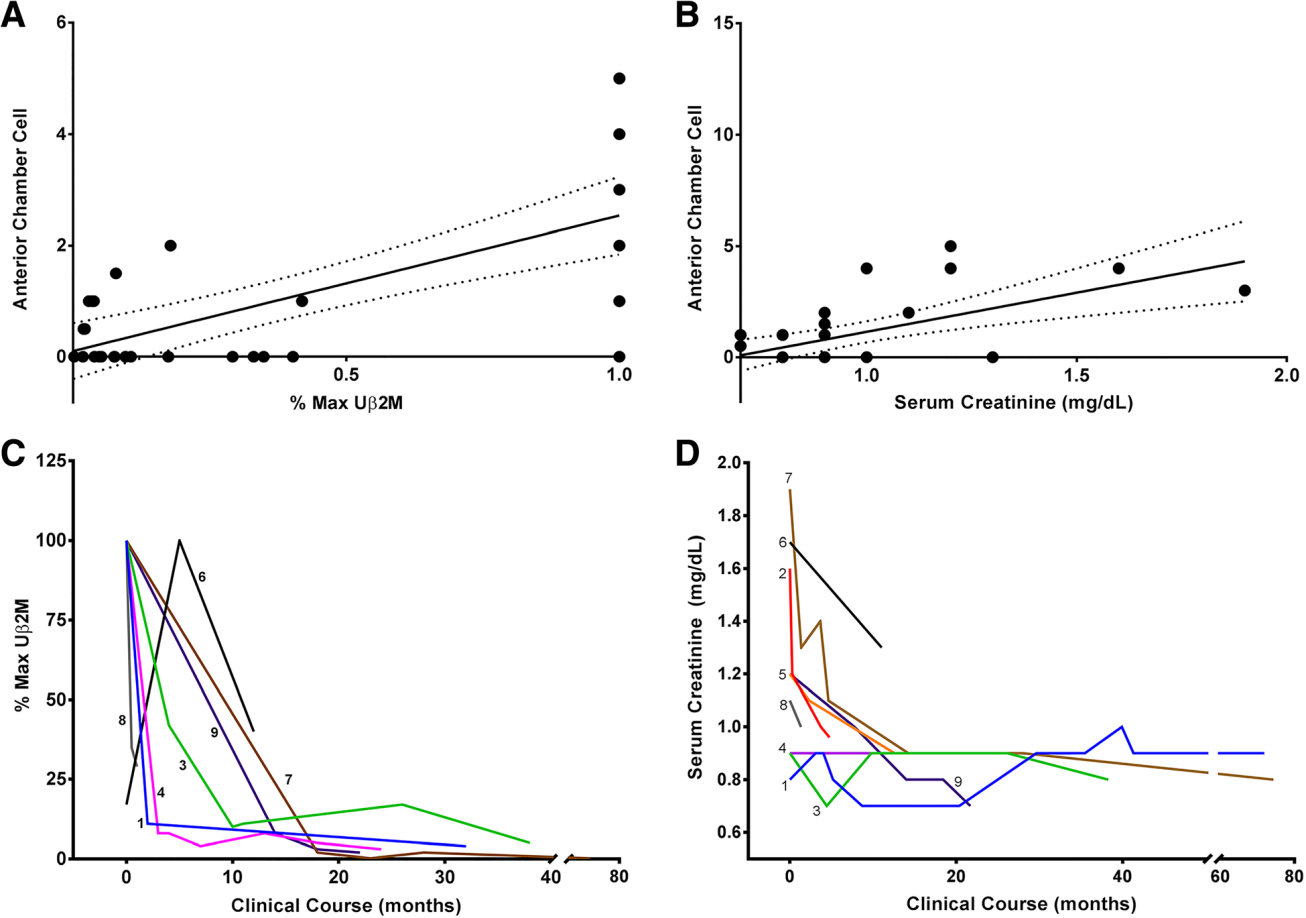
Urinary β2-microglobulin (Uβ2M) (*r* = 0.66, 95% CI 0.41–0.82) and serum creatinine (SCr) (*r* = 0.36, CI 0.02–0.63) correlate with uveitis activity. As Uβ2M decreases over time, from the maximum for individual patients, uveitis activity decreases. **a**, **b** Scatter plots for the % of maximum Uβ2M and corresponding uveitis scores and SCr and the corresponding uveitis scores for all patients combined over the study period. A linear regression line (solid) with 95% confidence intervals (dashed line) is shown. **c** The % of maximum Uβ2M for all seven patients (numbering corresponds to patients in Table 2) with repeated Uβ2M measurements decreased over the first 20 months for all seven patients with repeated Uβ2M measurements. **d** Serum creatinine normalized over the first year for patients 1 and 5–9. SCr was normal throughout the disease course for patients 3 and 4

Mean Uβ2M at presentation was 6536 μg/L (95% CI 2030–11,043), which was 40.9 times the upper limit of normal (normal < 160 μg/L). The median interval between the onset of uveitis and initial Uβ2M testing was 7 days (mean 26.4 [95% CI − 12.6–65.5]). There was a strong correlation between Uβ2M elevation and anterior chamber cell (*r* = 0.69, 95% CI 0.46–0.84) (Fig. 1a) and between Uβ2M elevation and flare (*r* = 0.65, 95% CI 0.39–0.81). There was no significant correlation with Uβ2M and anterior vitreous cell (*r* = 0.33, 95% CI − 0.12–0.66). In seven patients that had repeated Uβ2M measurements, Uβ2M trended downwards over the course of 1–2 years for most patients (Fig. 1c), and the mean final Uβ2M was 12.2% of the initial Uβ2M (95% CI − 2.4–26.8). Uβ2M had normalized in four patients at last follow-up. No significant correlation was detected between anterior vitreous cell and Uβ2M elevation (*r* = 0.32 95% CI − 0.12–0.66).

SCr was elevated at presentation in 7/9 patients (78%) with a mean SCr of 1.28 mg/dL (95% CI 1.00–1.56). SCr had a trend for weaker correlation with anterior chamber cell (*r* = 0.59, 95% CI 0.29–0.79) (Fig. 1b) and flare (*r* = 0.52, 95% CI 0.19–0.75) compared to Uβ2M, but the difference was not significant. There was no significant correlation with SCr and anterior vitreous cell (*r* = 0.21, 95% CI − 0.28–0.62). Over the disease course, SCr returned to normal within a few months and varied little after that time (Fig. 1d). SCr normalized in 6/7 patients. Mean final SCr was 0.93 mg/dL (95% CI 0.80–1.07).

Urine protein was detected on UA in just five patients: trace protein in four patients (44%) and 1+ protein in one patient (11%). No patient required dialysis. Table 2 shows the systemic laboratory workup and Mandeville criteria for individual patients. A renal biopsy was performed in three patients; all biopsies showed acute TIN. Four patients met Mandeville criteria for definitive TINU syndrome; five were probable. One patient had a history of gestational diabetes (Table 2, patient 6), which had resolved, but none of the other patients were diabetic.

**Table 2:** Initial laboratory data, disease course, and follow-up for individual patients

Patient	Age/sex	Uveitis location	Laboratory data										Renal biopsy	Mandeville criteria	Oral steroids	Follow-up (months)	Disease duration (months)	Remission
			ANA	ANCA	ACE	HLA-B27	RF	Lyme	Syphilis	Initial Uβ2M (μg/L)	Elevated SCr	UA protein						
1	12/M	A	−	–	–	–	–	–	–	2557	+	+	+	D	+	74	51.5	+
2	14/M	A	+	–	–	–	–	N	–	6745	+	–	+	D	+	7.7	7.7	–
3	15/M	A	–	–	–	–	–	–	–	1711	–	–	–	P	+	41.4	18	+
4	16/M	A	−	–	–	–	–	–	–	3093	–	–	–	P	+	26.3	26.3	–
5	18/F	P	–	–	–	N	N	–	–	3169	+	+	–	P	+	28.1	13.3	+
6	42/F	I	−	–	–	–	–	–	N	13,669	+	+	+	D	+	17	17	–
7	53/F	A	–	–		N	–	–	–	17,494	+	++	–	D	+	72.4	31.3	+
8	64/F	A	–	–	–	N	N	N	–	833	+	–	–	P	–	2.5	2.5	–
9	67/F	I	–	–	–	–	–	–	–	9557	+	+	–	P	+	59.8	46.7	+

*ANA* antinuclear antibody, *ANCA* antineutrophil antibody, *ACE* angiotensin-converting enzyme, *RF* rheumatoid factor, *Uβ2M* urine β-2 microglobulin, *SCr* serum creatinine, *UA* urinalysis, *F* female, *M* male, *A* anterior uveitis, *I* intermediate uveitis, *P* panuveitis, *N* not performed, *D* definite, *P* probable; + = trace protein on UA; ++ = 1+ protein on UA

### Treatment, follow-up, and ocular complications

All patients were treated with topical steroids, and oral steroids were used in 8/9 patients with a mean starting dose of 0.6 mg/kg/day (95% CI 0.5–0.8). Two patients (patients 1 and 2, Table 2) were also treated with mycophenolate mofetil by nephrology. Mean follow-up was 36.2 months (95% CI 15.6–56.9). At last follow-up, five (56%) patients were in remission. Relapse occurred once in two different patients. An exacerbation occurred in 7/9 patients within the first year. Patients had a mean final − 0.01 logMAR visual acuity (95% CI − 0.03–0.02), which was a significant improvement from presentation. Ocular complications are shown in Table 3.

**Table 3:** Ocular complications throughout the disease course

	Patients	Resolved
Posterior synechiae	1	1/1
Cataract	2	2/2
OHT/glaucoma	3^a^	1/3
Cystoid macular edema	1	1/1

*OHT* Ocular hypertension

^a^One patient had preexisting glaucoma

## Conclusions

TINU syndrome represents 1–2% of patients at tertiary uveitis centers [6, 7], which is a likely underrepresentation given the difficulty in recognizing acute TIN without appropriate laboratory testing early in the course of the condition [8]. Acute renal dysfunction (elevated SCr) may be self-limited in TINU syndrome, and traditional laboratory tests of renal function have been shown to rapidly normalize in TINU patients [11]. Depending on the timing of uveitis presentation, which precedes or follows TIN in 86% of patients, SCr may be normal and is not a useful marker for any residual, subclinical systemic disease [2]. Diagnosis represents only the first of many hurdles in the management of TINU syndrome, as uveitis tends to be chronic and relapsing, potentially leading to ocular sequelae. Most patients eventually end up with remission of uveitis, though the timing of remission varies [8, 13].

Multiple authors have proposed Uβ2M as a potentially useful diagnostic laboratory test in TINU syndrome [2, 8, 9, 13, 14]. In 1999, Takemura and colleagues found that Uβ2M was highly elevated in a cohort of patients with biopsy-proven TINU syndrome. Uβ2M decreased gradually over the course of the disease, remaining elevated longer than SCr. Mandeville then incorporated Uβ2M into the diagnostic criteria for interstitial nephritis and abnormal urinalysis, recommending testing if SCr was abnormal. Goda et al. later demonstrated that 92% of biopsy-proven TINU patients had elevated Uβ2M, while SCr, a marker of acute renal dysfunction, was elevated in just 25% [14]. In 2015, Hettinga et al. brought Uβ2M to the forefront as a diagnostic tool by demonstrating that Uβ2M has a positive predictive value (PPV) of 88% and a negative predictive value of 97% in detecting definite or probable TINU. When combined with in elevated SCr, the PPV increases to 100% [9].

We hypothesized that the role of Uβ2M may not be limited to that of a diagnostic test but may also provide information on the course of inflammatory activity in TINU syndrome. Our study is the first to report that Uβ2M levels correlate strongly with the activity of ocular inflammation over the course of TINU syndrome, and this correlation tends to be stronger than that of SCr. Whereas SCr rapidly normalizes, our cohort showed a decrease in Uβ2M over the course of their disease, which is similar to previously published data [11]. These data suggest that Uβ2M levels may guide the clinician, over time, in determining where the patient is in the disease course, ultimately helping with treatment decisions. Uβ2M levels may serve as an adjunct to observation of uveitis activity at the slit lamp in regard to when treatment for the uveitis may be discontinued. When the Uβ2M levels have normalized, our study suggests that the chance of the uveitis recurring after treatment is discontinued is far less than if the Uβ2M is still elevated. This is a unique finding in inflammatory ocular disease since the ability to anticipate recurrence is difficult upon stopping treatment in most forms of uveitis.

Our results also confirm that Uβ2M and SCr do not covary much in patients with TINU syndrome, which is in accordance with findings in biopsy-proven TINU syndrome [11, 14]. Uβ2M is passed almost completely through the glomerular membrane and then almost entirely reabsorbed in the normal proximal tubule. Thus, Uβ2M elevation in disease is primarily determined by proximal tubular dysfunction and is a marker for TIN [15]. Conversely, SCr is filtrated by the glomeruli as well as actively secreted by the proximal tubule and is considered a marker for nephropathy [16]. In TINU syndrome, the fact that Uβ2M can be elevated while SCr is normal, as well as the stronger correlation of Uβ2M elevation with uveitis activity, together seems to indicate that Uβ2M may be a more specific activity marker for ongoing subclinical systemic inflammation [15].

While our retrospective study makes a significant contribution to the small pool of literature on TINU syndrome, it has limitations. The study group was relatively small due to the rarity of the disease, and not all patients had complete data based on the retrospective nature of the study. Follow-up was variable due to the retrospective design of the study, which limits our understanding of relapse and remission. Uβ2M testing, when repeated, was done rarely and at variable time points. Data is insufficient to detect whether Uβ2M may predict relapse, treatment requirement, or complications.

In summary, we describe the novel finding of Uβ2M correlation with uveitis activity in TINU syndrome, implicating Uβ2M as an objective marker of systemic inflammatory activity and uveitis activity in patients with TINU syndrome. Uβ2M, especially in the setting of clinically inactive uveitis treatment, may provide information on where a patient is in their disease course and when a patient may safely taper or discontinue therapy. Additional study with prospective and more uniform intervals of Uβ2M measurement is recommended to further investigate the role of Uβ2M in TINU syndrome.

## Abbreviations

AC: Anterior chamber
ACE: Angiotensin-converting enzyme
ANA: Antinuclear antibody
ANCA: Anti-neutrophil antibody
CI: Confidence interval
EHR: Electronic health record
ICD: International Classification of Disease
IOP: Intraocular pressure
RF: Rheumatoid factor
SCr: Serum creatinine
SUN: Standardized uveitis nomenclature
TIN: Tubulointerstitial nephritis
TINU: Tubulointerstitial nephritis and uveitis
UA: Urinalysis
Uβ2M: Urinary β2-microglobulin
